# 800-kyr land temperature variations modulated by vegetation changes on Chinese Loess Plateau

**DOI:** 10.1038/s41467-019-09978-1

**Published:** 2019-04-29

**Authors:** Hongxuan Lu, Weiguo Liu, Hong Yang, Huanye Wang, Zhonghui Liu, Qin Leng, Youbin Sun, Weijian Zhou, Zhisheng An

**Affiliations:** 1 State Key Laboratory of Loess and Quaternary Geology, Institute of Earth Environment, Chinese Academy of Sciences, 710061 Xi’an, China; 2 CAS Center for Excellence in Quaternary Science and Global Change, 710061 Xi’an, China; 3 Laboratory for Terrestrial Environments, Department of Science and Technology, College of Arts and Sciences, Bryant University, Smithfield, RI 02917 USA; 4 Department of Earth Sciences, The University of Hong Kong, Hong Kong, China

**Keywords:** Climate sciences, Environmental sciences

## Abstract

The complicity of long-term land surface temperature (LST) changes has been under investigated and less understood, hindering our understanding of the history and mechanism of terrestrial climate change. Here, we report the longest (800 thousand years) LSTs based on distributions of soil fossil bacterial glycerol dialkyl glycerol tetraethers preserved in well-dated loess-paleosol sequences at the center of the Chinese Loess Plateau. We have found a previously-unrecognized increasing early and prolonged warming pattern toward the northwestern plateau at the onset of the past seven deglaciations, corresponding to the decrease in vegetation coverage, suggesting underlying surface vegetation or lack of has played an important role in regulating LSTs, superimposed on the fundamental global glacial–interglacial changes. Our results support that LSTs in semi-humid and semi-arid regions with little vegetation will be more sensitive to the anticipated global temperature rise, while improving vegetation coverage would reduce LSTs and thus ecological impacts.

## Abstract (web summary)

Modern observation indicates that vegetation cover could modulate land surface temperatures substantially. Here the authors demonstrate that such vegetation feedbacks could be clearly identified in the Chinese Loess Plateau land surface temperature records during past cool periods when vegetation cover was reduced.

## Introduction

Our current understanding of the patterns and processes of Quaternary temperature changes has heavily relied on studies of marine records, mainly due to ocean’s uniformity and large capacity of taking up excess heat. For example, the timing and magnitudes of sea surface temperature (SST) variations are recorded by the alkenone unsaturation index^1^, sea level and ice volume cycles are reflected in benthic oxygen isotope values (δ^18^O) from marine sediments^2^, and polar air temperatures can also be reconstructed using ice cores^3^. Even though these proxy-derived oceanic climate reconstructions have served as benchmarks for global climatic changes during the Quaternary, earlier response in marine realm, relative to ice volume changes, has been reported^4^, some of which has been linked to orbital obliquity control^4^. Although the classical terrestrial Devils Hole chronology^5^ has been revised to be more closely aligned with the marine record^6^, as the heterogeneity of land surface, such as topography and vegetation changes, can affect regional climate, direct comparisons between marine and land climate records remain to be difficult^7^. More terrestrial records are thus badly needed to better understand the general patterns and mechanisms of climatic changes on land. While cave records, elemental data, and lipid isotope signals offer records of monsoon intensity, and tree ring and pollen records can serve as short-term temperature proxies, long-term land paleotemperature reconstructions have been rare, primarily due to lacking of suitable proxies. Yet, a better understanding of terrestrial temperature variation is essential in deciphering global climatic changes, enhancing the development of climatic models, and predicting regional temperature variations on land. Particularly, amplified temperature changes due to global warming have become a typical feature at the middle and high latitudes of the Asian continent, with northern China, in particular^8^, having a profound impact on ecosystems, hydrological circulations, and agricultural production^9^.

**Fig. 1:**
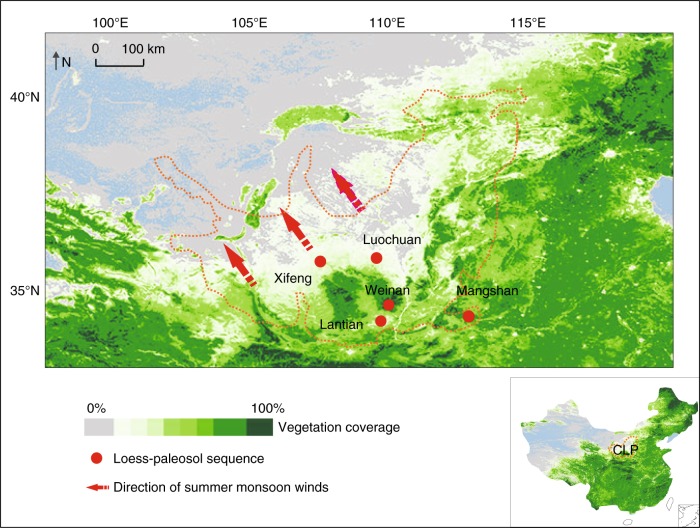
Geographic locations of Mangshan, Lantian, Weinan, Luochuan, and Xifeng loess*–*paleosol sections and modern vegetation coverage on the Chinese Loess Plateau. The base map is generated from the ArcGIS software. Vegetation coverage was calculated from the annual maximal Normalized Difference Vegetation Index of the SPOT VEGETATION satellite dataset as proposed in ref. ^50^. The area enclosed in orange line denotes the Chinese Loess Plateau. Modern vegetation coverage on the plateau decreases from southeast to northwest

The loess–paleosol sequences on the Chinese Loess Plateau (CLP) provide a valuable climatic archive with established and well-constrained chronological controls^10,11^. Various proxies have revealed the history of terrestrial climatic changes in relation to strengths of the East Asian monsoon (EAM) from 2400 to 2600 kyr ago (ka) to the Holocene^10^. Recent studies have shown that branched glycerol dialkyl glycerol tetraether (brGDGTs) (see Supplementary Fig. 1 for structures) derived from membranes of some heterotrophic bacteria are widely distributed in loess deposits, and the newly developed brGDGT proxy holds great potential for paleotemperature reconstruction^12–15^. Here we apply this method to two well-dated loess–paleosol sections, Luochuan (~800 kyr) and Xifeng (~600 kyr), at the center CLP (Fig. 1 and “Methods”) to reconstruct the longest terrestrial paleotemperatures, covering the last seven glacial–interglacial periods. A comparison with other geochemical records reconstructed from the same loess–paleosol sections, as well as marine/global records, reveals distinctive features of brGDGT-recorded land surface temperatures (LSTs) under the influence of vegetation coverage. We have found that such vegetation feedbacks are linked with the intensity of EAM, illustrating the importance and complicity of terrestrial vegetation coverage in affecting LST variations, at least in semi-humid and semi-arid regions. To assist our interpretation, we also present modern observations demonstrating progressively increased contrast between air and in situ LSTs toward desert regions.

## Results

### Modern LSTs vs. air temperatures

**Fig. 2:**
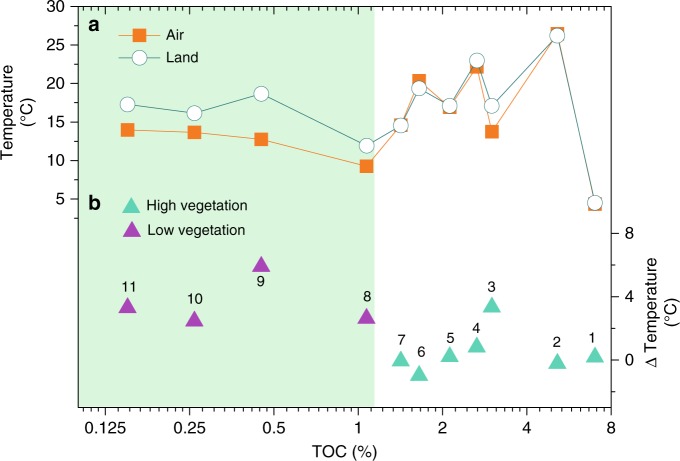
Measured land surface and air temperatures in bacterial growth seasons and the temperature difference against total organic carbon (TOC) contents at 11 locations in China. **a** Measured in situ land surface and air temperatures. **b** Temperature difference between measured in situ land surface and air temperatures. (1) Aershan, (2) Haikou, (3) Yijun, (4) Guilin, (5) Manchuanguan, (6) Taoyuan, (7) Luochuan, (8) Baiyunebo, (9) Wuyuan, (10) Yulin and (11) Dengkou. TOC content is used here to indicate vegetation cover. Bacterial growth seasons are defined as the period from March to November. The purple triangles represent the locations with low vegetation biomass, while green triangles represent the locations with high vegetation biomass. The highlighted low TOC (≤1.2%) interval indicates high temperature contrast in little vegetated regions. See Supplementary Fig. 2 for locations

We have retrieved in situ LSTs from 11 sites in China, shown together with modern air temperature data from nearby meteorological stations (Fig. 2). The two temperatures during bacterial growth seasons (March to November, “Methods”) diverge with reducing vegetation cover, indicated by total organic carbon (TOC) contents. The temperature difference could reach ~5 °C in little vegetated regions, while minimal in vegetated regions (Fig. 2). The LSTs from four selected meteorological stations, representing different vegetation zones in China (Supplementary Fig. 2), are quite similar to air temperatures in winter but increase faster in summer (Supplementary Fig. 3). The difference between the two temperatures increases with reduced vegetation cover, reaching maximal ~10 °C in July in the desert region (Moyu, Supplementary Fig. 3). Therefore, our modern observations strongly support that LSTs tend to be amplified as compared to air temperatures, due to poor vegetation cover. It remains intriguing whether such vegetation feedbacks could also be identified particularly during past glacial periods when vegetation coverage was substantially reduced in northern China^16,17^.

### LST evolution at Xifeng and Luochuan

**Fig. 3:**
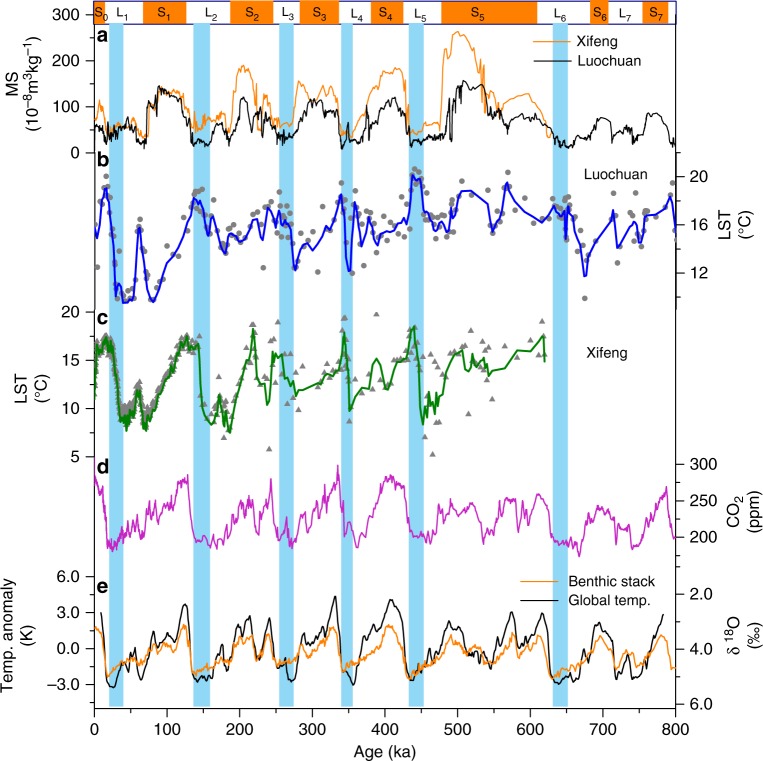
800-kyr land surface temperature (LST) records from Luochuan and Xifeng sections on the Chinese Loess Plateau, compared with onsite magnetic susceptibility and global/marine indicators. **a** Luochuan and Xifeng magnetic susceptibility (MS) records with major loess (L_1_–L_7_) and paleosol (S_0_–S_7,_ orange bars) units labeled. **b**, **c** LSTs from Luochuan and Xifeng, respectively, with their three-point moving averages. **d** Atmospheric CO_2_ from Antarctic ice core records^24^. **e** Reconstructed global sea surface temperatures^19^ and benthic δ^18^O stack^2^. The blue bars highlight intervals of early warming at Xifeng and Luochuan within glacial periods as indicated by other records. Initiation of LST rise led other indicators by ~7–20 kyr

Our new brGDGT-derived LST reconstructions (“Methods” and Supplementary Figs. 4 and 5 for chronology) extend our paleotemperature records to the past 800 kyr (Fig. 3), which allows us to determine the rate, timing, and magnitude of terrestrial temperature variations and their relationship to global climatic changes in uncovering possible influencing factors on land. Our Xifeng record expands the brGDGT analysis of this section by >500 kyr from its previous analysis^18^; and at the overlapped portion, our reconstructed temperature variations are congruent with the published, further demonstrating the robustness of our method. The reconstructed temperatures at Luochuan and Xifeng sections show a glacial–interglacial change of 4–10 °C, a range that is consistent with those from the Weinan section located at the southern tip of the CLP^14^. Our results indicate that LST range between glacial and interglacial periods is about 4–6 and 7–10 °C before and after 450 ka, respectively. The phenomenon of reduced amplitude before 450 ka is in good agreement with the Mid-Brunhes Event recorded in marine sediments and Antarctic ice^3,19–21^. Our 800-kyr records clearly show the dominance of the 100-kyr cycles, similar to magnetic susceptibility records from the same areas^11,22^, global temperature records^1,3,19^, and benthic δ^18^O stack^2^ (Fig. 3). This suggests that paleotemperature variations on the CLP were primarily controlled by glacial–interglacial cycles, consistent with previously established climatic variation patterns revealed by other loess-based proxies^10,23^ and in broad agreement with our understanding of periodicities due to orbital forcing^1,2,19,24^.

**Fig. 4:**
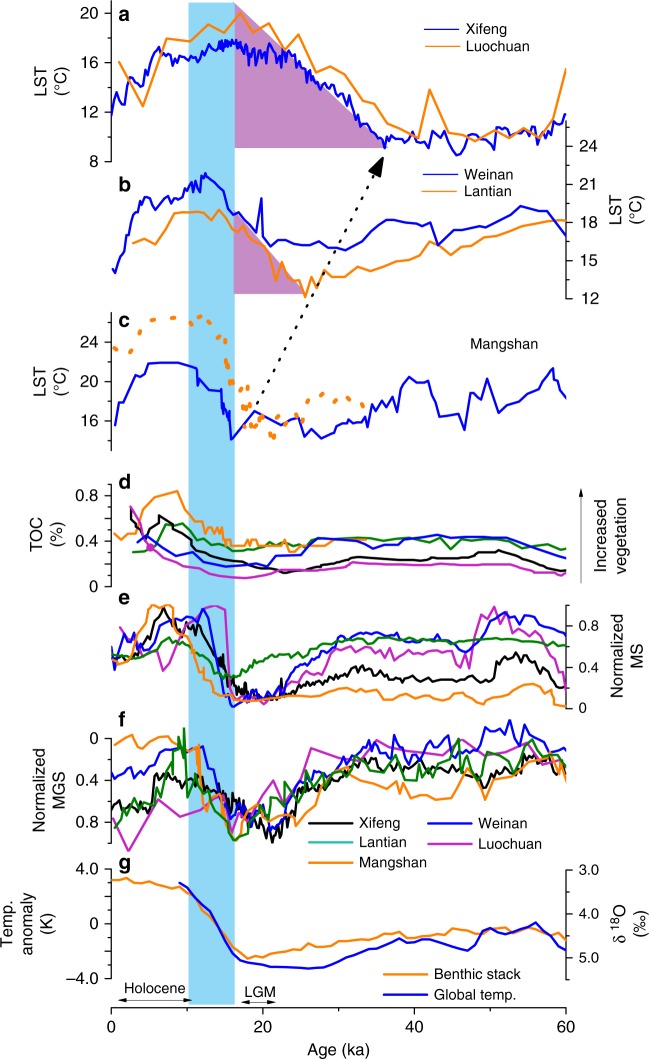
Spatial pattern of early land surface temperature (LST) rise on the Chinese Loess Plateau. **a** LSTs at Xifeng and Luochuan. **b** LSTs at Weinan^14^ and Lantian^15^. **c** LSTs at Mangshan (purple^12^, orange^69^). **d**–**f** Total organic carbon (TOC) content, normalized magnetic susceptibility (MS), and mean grain size (MGS) records at these sections (Mangshan, organge^69^; Lantian, olive; Weinan, blue^14^; Xifeng, black; Luochuan, purple). **g** Reconstructed global mean SSTs^7^ and benthic δ^18^O stack^2^. Earlier LST rise toward the northwestern Chinese Loess Plateau is highlighted with the black arrow and purple shaded areas, in line with reduced vegetation cover (lower TOC content in **d**) during the last glacial period. The last deglaciation into the Holocene, as indicated by the onsite MS/MGS and TOC content, and global/marine records (**d**–**g**) is highlighted with the blue bar. The early LST rise, relative to other proxies from the same sites, is a robust feature and irrespective of chronological uncertainty. See Fig. 1 for site locations

While our reconstructed temperatures are consistent among themselves and in tight correspondence with magnetic susceptibility/mean grain size (MS/MGS) variations derived from the same sections (Figs. 3 and 4), our new data show a couple of novel, and rather surprising, features. First, we have observed consistent offsets of the timing for the transitions from glacial to interglacial periods relative to the changing times in the onsite MS/MGS and TOC content, and ice volume and global temperature variations that are mainly derived from marine records (Figs. 3 and 4). Taking the Last Glacial period as an example, our Xifeng and Luochuan temperatures started to rise around 36 ka (Fig. 4); that is ~16 kyr in advance of the onsite MS/MGS and TOC changes, in addition to global ice volume maxima as indicated by global benthic δ^18^O and CO_2_ variations^2,24^. Our temperature records further indicate that such early warming at the center CLP occurred at nearly every glacial termination during the last seven glacial–interglacial periods spanning the past 800 kyr (Fig. 3). As a result, the timing of our reconstructed temperatures changes from Luochuan and Xifeng sections does not match with cold and warm events recorded in SST records or the marine benthic δ^18^O stack from previous studies^1–3^ (Fig. 3). Usually, during the Last Glacial Maximum (LGM) (25–15 ka) when continental ice sheets were at their greatest extension during the last glacial period, the lowest global temperatures of this period were recorded through marine proxies. However, this low temperature period is not reflected by our brGDGT-based reconstructions at either Luochuan or Xifeng (Fig. 4). Similarly, instead of recording the Holocene thermal maximum of a relatively warming period between ~11 and 5 ka, our reconstructed temperature curves show a much longer warming duration between ~20 and 5 ka at the center CLP.

Second, compared with most marine proxy-derived temperatures and MS/MGS records from the same sites, our reconstructed temperatures record a longer time period when transitioning from glacials into interglacials. During the last transition, instead of ~10 kyr registered in SST and MS/MGS records, our data recorded a prolonged time period of ~20 kyr (Fig. 4) for glacial termination. The observed gradual glacial termination in our records is inconsistent with the much rapid termination of ice ages that have been documented in most marine^19^, ice core^3^, and cave stalagmite records^25^ over the past 800 kyr.

### Spatial pattern of early warming

We compare our LSTs with previously published brGDGT data from other three CLP sections: Mangshan (34° 56′ N, 113° 22′ E), Lantian (34° 12′ N, 109° 12 ′E), and Weinan (34° 21′ N, 109° 32′ E) (see Fig. 1 for locations) over the past 60 kyr (Fig. 4). An obvious increasing trend of early warming times from the southeastern Mangshan to central Luochuan and Xifeng sections can be well identified. The onset of warming began around ~36 ka at Luochuan and Xifeng, compared with ~23 ka at Lantian and Weinan, and then decreased to 15–20 ka at Mangshan (Fig. 4). In contrast, MS/MGS records from those same sections, as well as TOC contents to indicate vegetation changes here, show largely synchronous changes, with transitions typically taking place between ~20 and 10 ka, consistent with the global pattern of the last deglaciation (Fig. 4). In other words, the early warming reflected in the brGDGT-derived LSTs, relative to their onsite MS/MGS and TOC changes, is a robust feature, irrespective of any chronological uncertainty.

## Discussion

The chronology of loess sections is commonly established based on the correlation of MS/MGS with marine benthic δ^18^O (ice volume) or orbital parameters (“Methods”), and constrained with paleomagnetic data when the studied section is sufficiently long. Consequently, MS/MGS variations, proxies for East Asian summer and winter monsoon, respectively, from various sections on the CLP are essentially synchronous on glacial–interglacial timescales (Supplementary Fig. 6), a fundamental tenet in loess studies. Sediment hiatus, if presented, could affect the correlation-based chronology. A 4–5-kyr hiatus in the typical loess deposits has been reported in several studies based on independent quartz optically stimulated luminescence (OSL) dating technology, indicating that the loess record is not continuous over millennial timescales, and this hiatus may be forced by a period of strengthened East Asian winter monsoon and less vegetation cover at the investigated sites^26–28^. However, at other sites sediment hiatus is not detectable, suggesting that dust accumulation for the last glacial loess is generally continuous at millennial timescales on the CLP^29–31^. The inferred hiatus typically occurred at the paleosol–loess boundary, where processes of pedogenesis and carbonate leaching could both affect the estimates of OSL ages^26,32,33^. Some of the inferred hiatuses are actually an artifact of inaccurate determination of OSL ages^33^. Therefore, sediment hiatus over the major part of the CLP including the classical Xifeng and Luochuan sections (except desert margins^34^) might be minimal over millennial timescales^29,32,33^.

In order to further evaluate chronological uncertainty, we compare age discrepancies between correlation-based and high-resolution OSL-derived chronologies from Weinan^35^, Xifeng^36^, Luochuan^37^ and Jingyuan^23^ sections. Over the past 60/130 kyr, the age difference is within 5 kyr for most periods and <10 kyr in extreme cases (Supplementary Fig. 7). Consequently, the MS/MGS changes still took place between ~10 and 20 ka, with either of the two chronologies (Supplementary Fig. 8). Therefore, the high-resolution OSL ages from the last glacial period strongly support the fidelity of the correlation approach that spans the last seven glacial–interglacial cycles in the present study.

We also examined the early signals in our temperature records, relative to the onsite MS variations from Xifeng, Weinan, Lantian, and Mangshan loess profiles, in depth domain without transforming into ages. The offset in depth between LSTs and MS, supporting the early warming, becomes larger from the southeast to northwest of the CLP (Supplementary Fig. 9). Considering loess deposition rate at Xifeng (~3.9–22.5 cm/kyr, average 11.5 cm/kyr^36^) is lower than that at Mangshan, ~26 cm/kyr^38^, the spatial pattern of early warming would be more apparent in time domain. Therefore, the early warming relative to MS/MGS changes and the spatial pattern of early warming on the CLP reported in this study are largely unaffected by chronological uncertainty, based on our assessment of sediment hiatus, comparison of the two chronologies, and examination of the signal offset in depth domain.

Global and local studies of modern processes have shown that the distribution of brGDGTs corresponds well with temperature^39–43^, which can be used to reconstruct paleotemperature changes on land^12,13,39^, although the specific biological source of brGDGTs has not been identified. Currently brGDGT-based temperature reconstructions are generally calibrated against mean annual air temperature at both global and regional scales due to the lack of soil temperature data^39–43^. However, we argue that brGDGT-based temperature reconstructions from these loess sections should best be interpreted as near-ground LSTs during bacterial growth seasons since bacterial production of brGDGTs on the CLP occurs during warm, wet seasons^39^. A recent investigation using an independent set of 20 soil samples collected from temperate area of northern China has also demonstrated that brGDGT indices correlate strongly with growing season (March to November) soil temperatures but respond weakly to winter soil temperatures^43^.

Although responding to air temperatures, LST variability registered in brGDGTs produced by bacteria living within soil layers is more directly influenced by the near-ground climate. The near-ground climate may substantially differ from the air layer (defined as a height of ~1.5 m above the level of the ground), since when ground surface is approached, many atmospheric elements change rapidly^44^ and can be further modified by soil physical parameters (such as albedo, texture, and moisture)^45^. This distinction is important as land surface barriers impact near surface radiation and alter surface energy balance as well as boundary development in the way of influencing soil and near surface temperatures. We therefore interpret brGDGT-based temperatures to indicate LSTs during bacterial growing seasons on the CLP.

Although brGDGT-producing bacteria may survive in deeper soils, brGDGTs produced in surface soil should predominate. The lifestyle of brGDGT-synthesizing bacteria is heterotrophic^46^, and thus surface soil is more suitable for bacterial growth due to available organic matter and oxygen. A stable isotope probing experiment has also demonstrated that brGDGT-producing bacteria in peat are more active in the aerobic acrotelm than the anoxic catotelm^46^. Further, brGDGTs concentrations in surface soil (0–5 cm) are generally much higher than those in deeper soil (5–30 cm) at Xifeng, Lantian, and Mangshan (Supplementary Fig. 10), supporting that brGDGTs are mainly produced in the surface soil layer, at least on the CLP.

We also examined downcore variations of LST, brGDGT concentration, brGDGT concentration normalized by TOC content, TOC content, and MS in the Xifeng profile. Changes in all these contents correspond well with MS, while LST changes are obviously ahead of them (Supplementary Fig. 11). Generally, higher LSTs correspond with lower brGDGT concentrations. Therefore, the higher LSTs unlikely result from downward migration of brGDGTs produced at a later time or in situ modern production in deeper soils. Bioturbation and diagenesis, although affecting GDGT concentrations, have neglected effects on GDGT temperature proxies^47–49^. Therefore, the early warming observed in our temperature records is unlikely due to the alteration of brGDGT distributions after burial in loess–paleosol sequences or chronological uncertainty as assessed above and requires a climatic interpretation.

Modern investigations show that vegetation coverage on the CLP gradually decreases from southeast to northwest (Fig. 1). For instance, remote sensing data^50^ indicate that vegetation coverage decreases from 50–60% at Mangshan to 20–30% at Xifeng in 1999. Modern TOC content also decreases toward the northwest CLP^51^. However, past vegetation coverage reconstruction remains difficult. Pollen records suggest that during the LGM, temperate grassland, xerophytic shrubland and desert dominated northern China, including the CLP (Supplementary Fig. 12). Several sections on the CLP^17^ reveal the presence of desert/steppe vegetation on the plateau during the LGM, with more abundant Poaceae at the southwest. However, as pollen records largely represent large-scale regional vegetation changes, not at a particular locality, we thus here use TOC content as an indicator of vegetation cover at those study sites, previously proposed to reflect vegetation history and biomass variations on the CLP^52^.

TOC contents at Xifeng, Luochuan, Weinan, and Mangshan over the past 60 kyr show generally synchronous changes with the MS, as well as pollen-inferred vegetation changes in northern China and global climate but clearly decouple from the onsite LST changes (Fig. 4, Supplementary Fig. 12). That is, the TOC-inferred vegetation cover gradually decreased and reached the lowest value during the LGM and then increased gradually in parallel with the monsoon intensity indicators such as MS/MGS (Fig. 4, Supplementary Fig. 12). The TOC-inferred vegetation variations on the CLP also indicate a decrease in vegetation coverage from southeast to northwest, in parallel with the reduction of EAM influence. As such, TOC contents at Luochuan and Xifeng sections remained <0.3% throughout the last glacial period until the early Holocene, while being higher toward the southeastern CLP (Fig. 4). Previous published δ^13^C-based precipitation reconstructions on the CLP are in high level of congruence with variations of TOC contents^53^, providing additional support for the EAM-controlled vegetation gradient on the CLP. It can thus be inferred that, during the past glacial period, particularly LGM, desert/steppe vegetation dominated the center CLP, such as Xifeng and Luochuan, whereas substantial vegetation cover still existed at Mangshan, the southeast CLP. The spatial trajectory of vegetation coverage change is also consistent with the pattern of early warming times recorded in our reconstructed LSTs (Fig. 4).

These distinctive features captured in our LST reconstructions (Figs. 3 and 4) can be explained by a combination of better understanding of the nature of the brGDGT proxy and considering the vegetation feedback on the CLP under the EAM influence, offering an opportunity to tease out the complicity of continental temperature reconstructions in Asian semi-humid and semi-arid regions. Because of the nature of the brGDGT proxy mentioned above, the influence of local vegetation variations on LSTs during bacterial growing seasons may be more pronounced on the relatively dry CLP, causing a large deviation from air temperatures. These factors become even more apparent when we closely examine our LST records in the context of overall features on the CLP.

Previous studies on loess–paleosol sections have demonstrated the importance of solar insolation on temperature variations on the south CLP, in addition to other potential drivers (e.g., atmospheric CO_2_ and Southern Hemisphere cooling)^12,39^. However, the obvious mismatch observed in our LST records indicates the existence of other factor(s) at working in modulating LSTs on the CLP. These five loess–paleosol sections are quite close geographically (~600 km between Mangshan and Xifeng) and have minor latitudinal differences, only ~1.5° from the southernmost Lantian to the northernmost Luochuan. Therefore, it is unlikely that the time difference in temperature rise was due to local insolation affected by orbital parameters or variations in greenhouse gas concentrations. The fact that the spatial distribution of these sections and the advancement of warming times correspond well with the reduction of EAM influence suggests a connection between these LST changes and a powerful covariant factor that is controlled by the EAM on the CLP.

We suggest that the EAM-controlled vegetation variations on the CLP have exerted an important influence on LST variations, and have thus tested this hypothesis in both spatial and temporal perspectives. Vegetation feedback on climate has been previously noticed^54,55^. Vegetation’s influence on air temperatures is believed to be relatively weak (probably <2 °C), although modeling experiments have demonstrated that vegetation change is an important mechanism contributing to climate configurations during the mid-Holocene and the last interglacial period^55^. Such vegetation feedbacks can be intensified in soil layers in semi-humid and semi-arid regions, causing larger and amplified LST variations registered in brGDGT proxies, primarily due to heat transfer and influx balanced by the interaction between soil and vegetation^54,56^. The critical role that local vegetation variations have played in altering LSTs has also been illustrated in the following cases. A 12-month document recording daily soil temperatures from forest, woodland, and grassland sites in the Meru National Park in Kenya has well demonstrated an obvious influence of vegetation types on soil surface temperatures^54^. While air temperatures remain similar in the park, open areas have daily high surface ground temperatures up to 25 °C higher than those at the nearby well-shaded areas, due to higher light intensity and lower soil moisture without woody canopy coverage^54^. The measured in situ surface soil temperatures obtained by temperature loggers placed at 11 soil study sites (Fig. 2), as well as the 4 selected meteorological stations representing different vegetation zones in China (Supplementary Fig. 3), also show that the difference between LSTs and air temperatures increases with reducing vegetation coverage, especially during soil bacterial growing seasons.

As the way solar radiation is absorbed in soils varies, different underlying surfaces may affect the distribution of energy between the atmosphere and surface soil^57^. Vegetation changes can directly or indirectly influence spatial and temporal variations of soil temperatures in a number of ways through physiological activities underground and changing heat fluxes on and above ground through changing shading (measured by means such as leaf area index, projected leaf area per unit of ground area), ground litter stores, and soil and air moisture^56^. Generally, two opposing vegetation feedback effects may occur on heat fluxes: warming through increased energy absorption (i.e., relatively low albedo) and cooling through increased evaporation^58^. The net impact of vegetation feedback (warming or cooling) that can be registered in the brGDGT-based proxy depends on which influence is predominant. Normally, loess stages developed during the cold-dry glacial periods with relatively low vegetation coverage. Cold air temperatures may have prevailed on the whole CLP as suggested by pollen records^16,17,59^. During these periods, however, the poor vegetation and low soil moisture facilitate more efficient heat absorption to warm up soil layers. This may explain why our brGDGT-derived LSTs are insensitive to the extremely cold air temperatures during the LGM, when the minimal vegetation cover resulted in a maximal difference between air temperatures and LSTs. All of our observed early LST warmings occurred within loess stages at regions with low TOC contents and soil δ^13^C-based precipitation^53^, corresponding to poor vegetation coverage toward the northwestern CLP. The longer warming duration and lower rates of temperature transitioning from glacials into interglacials recorded in our LST reconstructions at the center CLP appear to reflect a different land surface energy balance modulated by a significant vegetation feedback, resulting in the insensitivity of LST to air temperature changes. The large deviations (mean 6.0 °C) between modern land surface and air temperatures observed in regions with little vegetation cover (Supplementary Fig. 3), similar to the temperature variations between glacials and interglacials (4–10 °C) (Fig. 3), further support that, in semi-humid and semi-arid regions, the brGDGT-based LSTs could have increased to a higher level, while air temperatures remained low during glacial periods, corresponding to the global signal.

The analysis of our LST records spanning the past 800 kyr in loess–paleosol sequences at the center CLP leads us to conclude that surface vegetation variations may have played a substantial role in regulating near-surface land temperatures. The complicity of interpreting terrestrial temperature records is manifested at our CLP case, while not fundamentally challenging the orbital (“Milankovitch”) theory of the Ice Ages. Well-constrained regional LST signals, such as the ones from our brGDGT-based reconstructions, can enrich our understanding of seemingly nonsynchronous climatic variations on land due to surface dynamics. This long neglected mechanism of terrestrial paleotemperature changes opens new windows for studies of terrestrial climate change, as well as improving projections of terrestrial climate. Our results confirm that LST changes in semi-humid and semi-arid regions with little vegetation cover are more sensitive to global warming and also reinforce one of the obvious, but less appreciated, beliefs that improving vegetation coverage on the CLP would help mitigate ecological impacts under the projected warming trend by reducing near-surface temperatures.

## Methods

### Materials

The Luochuan section (35° 47’ N, 109° 26’ E) lies at the center of the CLP (Fig. 1), with a current mean annual temperature (MAT) of 9.6 °C and a mean annual precipitation (MAP) of 592 mm (based on China Meteorological Administration climate records during 1981–2010, http://www.cma.gov.cn). The total thickness of the Luochuan loess section is 138 m. We collected samples from the upper 60 m, including 7 consecutive loess layers (L_1_–L_7_) and paleosol layers (S_0_–S_7_) spanning the past 800 kyr. A total of 270 samples were collected and analyzed from the Luochuan section at 25 cm intervals, giving an average sampling resolution of 3 kyr.

Another loess–paleosol sequence from the Xifeng section (35° 42’ N, 107° 38’ E), spanning the past 600 kyr, was also sampled and analyzed. The Xifeng section lies also at the center CLP, ~250 km away from the Luochuan section, with its current MAT and MAP of 9.2 °C and 480 mm, respectively. Samples were taken from two sites at Xifeng: the 16 m eastern site covers the S_0_ (Holocene) and S_1_, whereas the nearby 40 m western site (3 km away) extends the sequence continuously down to S_8_ (Marine Isotope Stage 15). A total of 363 samples with an approximate sampling resolution of 0.4 kyr were taken from the eastern site, while a total of 145 samples with a resolution of 3.3 kyr were taken from the western site.

### Chronology

The chronology of this study is based on the generally accepted correlation scheme of loess–paleosol sequences with marine benthic δ^18^O records (Supplementary Fig. 4), with loess (L_1_-L_7_) accumulated during glacial periods and paleosols (S_0_–S_7_) formed during interglacial periods. Weighted interpolation is employed following the established grain size-based age model^60^.

$$T_m = T_1 + \left( {\mathop {\sum }\limits_{i = 1}^m a_is_i} \right)\left( {T_2 - T_1} \right)\left( {\mathop {\sum }\limits_{i = 1}^n a_is_i} \right)$$

where *T*_1_ and *T*_2_ are age control points, respectively; *a*_*i*_ is the accumulation rate at level *i*, which is assumed to be proportional to MGS; *n* is the total sampling level between *T*_1_ and *T*_2_; and *m* is the sampling level at *T*_1_ and *T*_2_. The age control points over the past 800 kyr were transferred from MIS boundaries^2^ by correlating them with interglacial palaeosol boundaries^60,61^. Between control points, interpolation weighted by grain size was used to derive the chronology (Supplementary Fig. 4).

An alternative chronology for the Xifeng and Luochuan sections over the past 60/130 kyr, where high-resolution OSL ages are available^36,37^, is constructed in order to assess chronological uncertainty. We used the BACON software^62^ to derive the OSL-based chronology. Then the OSL chronology was mapped to our depth scale by comparison of loess–paleosol stratigraphy and MGS variations (Supplementary Fig. 5). Similarly, the correlation-based and OSL-derived chronologies were constructed for the Weinan and Jingyuan sections, where high-resolution OSL ages are also available^23,35^. The age differences between the two chronologies for the four sections are shown together in Supplementary Fig. 6.

Given the established chronological framework on the CLP^60^ and our robust age model that is verified by independently dated records (Supplementary Fig. 6), we view that our observed early rise of LSTs is highly unlikely due to chronology uncertainty. Although different timescales are available for the Quaternary loess of China^63,64^, these chronologies are generally consistent, at least on glacial–interglacial timescales, over the past 800 kyr^65^. Major sources of age uncertainty in our age model can be from the determination of age control points and the interpolation methods, since a transition period exists between high and low MGS values and the stratigraphic boundary defined by the MGS signal may be time-transgressive, depending upon the rates of weathering and dust deposition at each section^66^. However, comparison between the tie points and mid-points of the intervals shows that differences between them are 1.58 ± 1.84 kyr (*n* = 1083) for the Luochuan section^61^, whereas comparisons between simple linear and weighted interpolations are 1.22 ± 1.11 kyr (*n* = 1083) for the Luochuan section^22^. Therefore, although this correlation approach probably bears some uncertainties of <2 kyr, such uncertainties will not affect our main conclusion based on at a much larger period (i.e., >10-kyr difference).

### MS, MGS, and TOC analysis

MS was measured using a MS_2_ Bartington magnetic susceptibility meter at the Institute of Earth Environment, Chinese Academy of Sciences. For TOC analysis, the carbonate fraction of homogenized materials was removed using 2 mol/l HCl, then rinsed thoroughly four times in deionized water and dried. For MGS analysis, all samples were pretreated to remove organic matter and calcium carbonate using 30% hydrogen peroxide (H_2_O_2_) and 6 N hydrochloric acid (HCl), respectively^67^, and then dispersed by ultrasonification with 10 ml 10% (NaPO_3_)_6_ solution^23^. Grain size distribution was carried out on a Malvern 2000 laser instrument with an analytical error of within 2% at the Institute of Earth Environment, Chinese Academy of Sciences. TOC was measured on a Vario EL III elemental analyzer (Hanau, Germany), with an error <0.2%^68^.

### GDGT analysis

For GDGT (for structures, refer to Supplementary Fig. 1) analysis, homogenized materials (~25 g) were extracted with dichloromethane (DCM):methanol (9:1) using an accelerated solvent extractor (ASE 350) at 100 °C and 1500 psi. This process was conducted in three cycles with 5 min of heating followed by 5 min of static extraction. The extracts were dried under a gentle stream of N_2_ and isolated with a silica gel column with 25 ml of DCM:ethyl acetate (3:7, V/V). The extracts were dried under N_2_, re-dissolved in hexane:isopropanol (99:1, V/V), and filtered over a 0.45-μm polytetrafluoroethylene filter before analysis.

The branched GDGTs were analyzed using a Shimadzu liquid chromatography triple quadruple mass spectrometry system (LC-MS 8030) with an autosampler and Labsolutions manager software. Detection was achieved in an atmospheric pressure chemical ionization chamber with selected ion monitoring at *m*/*z* 1050, 1048, 1046, 1036, 1034, 1032, 1022, 1020, and 1018. The brGDGTs were quantified from integrated peak areas of the [M + H]^+^ ions and compared with the C_46_ internal standard. Source conditions were listed below: interface voltage 4500 V, interface temperature 350 °C, drying gas (N_2_) flow 5 L/min, Neb gas flow 2.5 L/min, and heat block temperature 250 °C. The injection volume was 50 μL. Separation of brGDGTs was achieved with two coupled Inertsil silica columns (250 mm × 4.6 mm, 3 μm). GDGTs were separated isocratically for 85 min with 95% *n*-hexane and 5% isopropanol, at a flow rate of 0.6 mL/min. After each analysis, the column was cleaned by flushing using 10% *n*-hexane/90% isopropanol for 20 min.

To derive land temperature from brGDGT distributions in the loess, MAT_mr_ were calculated over the past 130 kyrs using the following equations according to the most recent proxy calibration^42^:

$$\begin{array}{l}{\mathrm{MAT}}_{{\mathrm{mr}}} = 7.17 + 17.1 \times \left[ {{\mathrm{Ia}}} \right] + 25.9 \, \times \left[ {{\mathrm{Ib}}} \right] + 34.4 \, \times \left[ {{\mathrm{Ic}}} \right]\\ - \, 28.6 \, \times \left[ {{\mathrm{IIa}}} \right]\left( {n = 222,r^2 = 0.68} \right)\end{array}$$

where the fractional abundance of Ia, Ib, Ic, and IIa was relative to the sum of all brGDGTs. Owing to the very low concentration of brGDGTs in some samples, MAT were calculated using the three most abundant and omnipresent brGDGTs Ia, IIa, and IIIa, in the form of the Multiple Linear Regression Simple index (MAT_mrs_)^42^ over the past 130–800 kyrs:

$$\begin{array}{l}{\mathrm{MAT}}_{{\mathrm{mrs}}} = 5.58 + 17.91 \times \left[ {{\mathrm{Ia}}} \right] - 18.77 \, \times \left[ {{\mathrm{IIa}}} \right]\\ \left( {n = 231,r^2 = 0.62} \right)\end{array}$$

where the fractional abundance of Ia and IIa was relative to the sum of Ia, IIa, and IIIa. Over the past 130 kyrs, the two calibrations give very similar results (Supplementary Fig. 11).

The average analytical reproducibility of the MAT_mrs_ based on duplicate injections of a selected set of loess–paleosol samples is ca. 0.5 °C. A laboratory internal standard (lacustrine sediment) was also injected after every 30 samples in order to check the repeatability of the sample test and the average analytical reproducibility of the MAT_mrs_ is <0.3 °C.

To further verify the reliability of our method, we recalculated paleotemperature using our current method from the Lantian loess–paleosol sequence over the past 60 kyrs with the one that was reported previously in literature^15,18^ and found that the structure of the two curves is in good agreement, demonstrating the reproducibility of our analytical method and the relationship between brGDGT distribution and temperature reconstruction.

It should also be noted that there exist several proxies and calibrations for the paleotemperature reconstruction using brGDGT distributions. Although absolute values of reconstructed temperatures may vary, the structures of paleotemperature curves reconstructed by different proxies and calibrations are generally similar for the same section, especially for the timing of deglacial warmings^15,18,39^. Therefore, such uncertainties will not affect our major findings.

### Direct measurements of in situ LSTs

To better understand possible controlling factors, we measured in situ LSTs in one whole year at 11 sites along a roughly south–north transact in China, covering various latitudinal, topographical, vegetation, and climatic variables. The geographic coordinates of these locations are as follows: Haikou (19° 56’ 40” N, 110° 12’ 10” E), Guilin (109° 58’ 14” E, 24° 59’ 05” N), Taoyuan (28° 49’ 25” N, 111° 30’ 08“E), Manchuanguan (33° 09’ 39“N, 110° 09’ 09” E), Yijun (35° 23’ 20” N, 109° 07’ 58” E), Luochuan (35° 42’ 42” N, 109° 25’ 02” E), Yulin (38° 33’ 5” N, 109° 39’ 28” E), Dengkou (40° 09’ 14” N, 106° 54’ 4” E), Wuyuan (41° 18’ 35” N, 108° 25’ 30” E), Baiyunebo (41° 24’ 35” N, 109° 57’ 47” E), and Aershan (41° 31’ 31” N, 119° 25’ 24” E) (See Supplementary Fig. 2 for the locations). Temperature loggers (Thermochron iButton DS1922L-F5#) were deployed at ca. 5 cm soil depth for these sites to record LSTs at time intervals of every 2 h. After 1 year of burial, the temperature loggers were retrieved and the measured LST was exported and analyzed. The defined temperature in bacterial growth seasons was averaged over March to November.

## Supplementary information

Supplementary Information

## Data Availability

All relevant data that support the findings of this research are available from the corresponding author on request.
