# Supplementary material for: 800-kyr land temperature variations modulated by vegetation changes on Chinese Loess Plateau
Source: Nat Commun. 2019 Apr 29;10:1958. doi: 10.1038/s41467-019-09978-1 (PMC6488643; doi:10.1038/s41467-019-09978-1)
Supplement: Supplementary file 1 — Supplementary Information [file 41467_2019_9978_MOESM1_ESM.pdf]

Supplementary Information

800-kyr land temperature variations modulated by vegetation changes on Chinese Loess Plateau

Lu et al.

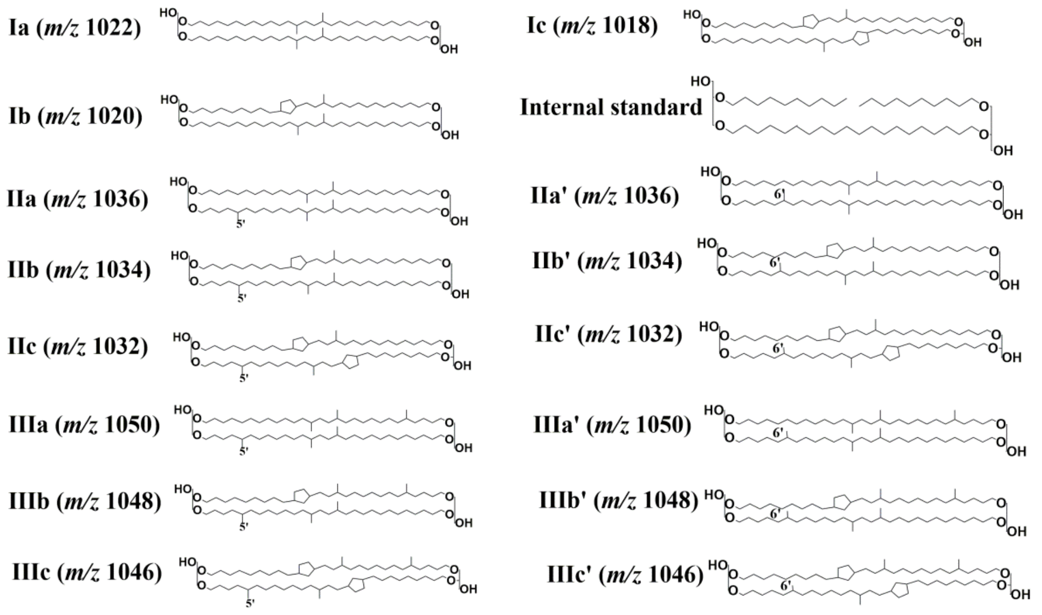

Supplementary Figure 1 Structures of branched glycerol dialkyl glycerol tetraethers and internal standard. Branched glycerol dialkyl glycerol tetraethers (brGDGTs) Ia, Ib and Ic are referred to as 'tetra-methylated,' brGDGTs IIa, IIa', IIb, IIb', IIc and IIc' are referred to as 'penta-methylated,' brGDGTs IIIa, IIIa', IIIb, IIIb', IIIc and IIIc' are referred to as 'hexa-methylated' brGDGTs (modified after ref. 1).

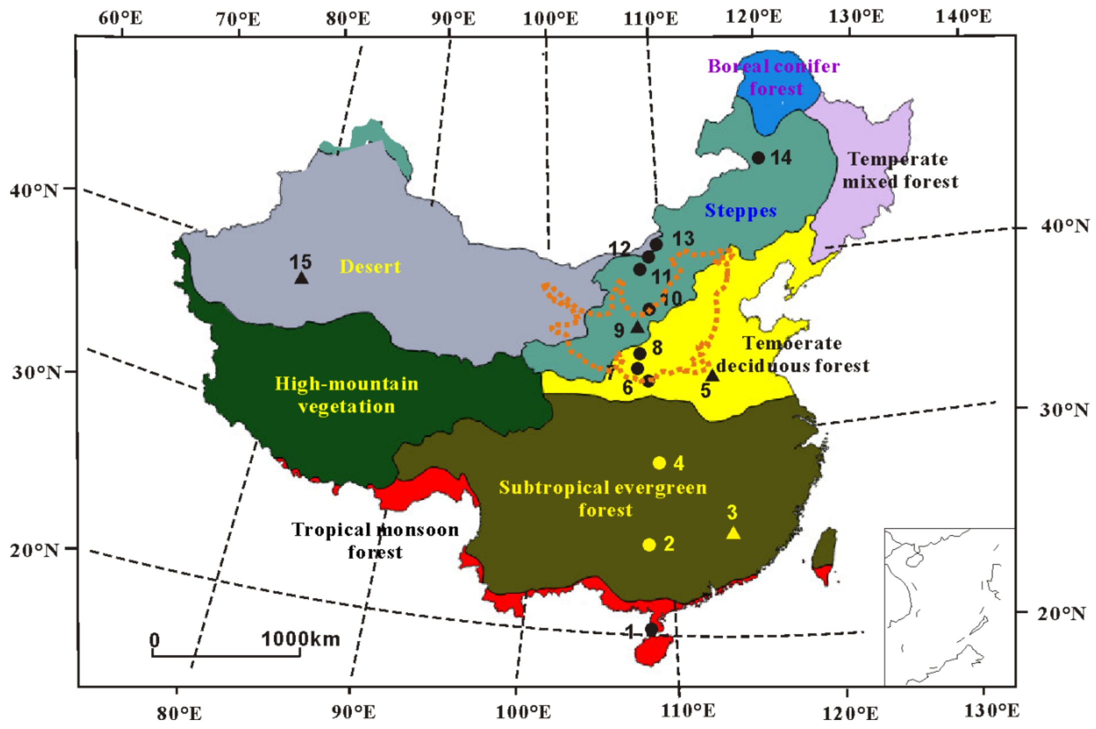

Supplementary Figure 2 Geographic locations of examined temperature measurements superimposed on the vegetation map of China. Four selected meteorological stations (marked with ▲), representing different vegetation zones in China, are Zengcheng (3), Zhengzhou (5), Jingyuan (9), and Moyu (15). Eleven sampling sites for *in situ* soil surface temperature measurements (marked with ●) are Haikou (1), Guilin (2), Toayuan (4), Manchuanguan (6), Yijun (7), Luochuan (8), Yulin (10), Dengkou (11), Wuyuan (12), Baiyunebo (13) and Aershan (14). The area enclosed in orange line denotes the Chinese Loess Plateau. The vegetation map of China is modified after ref. 2.

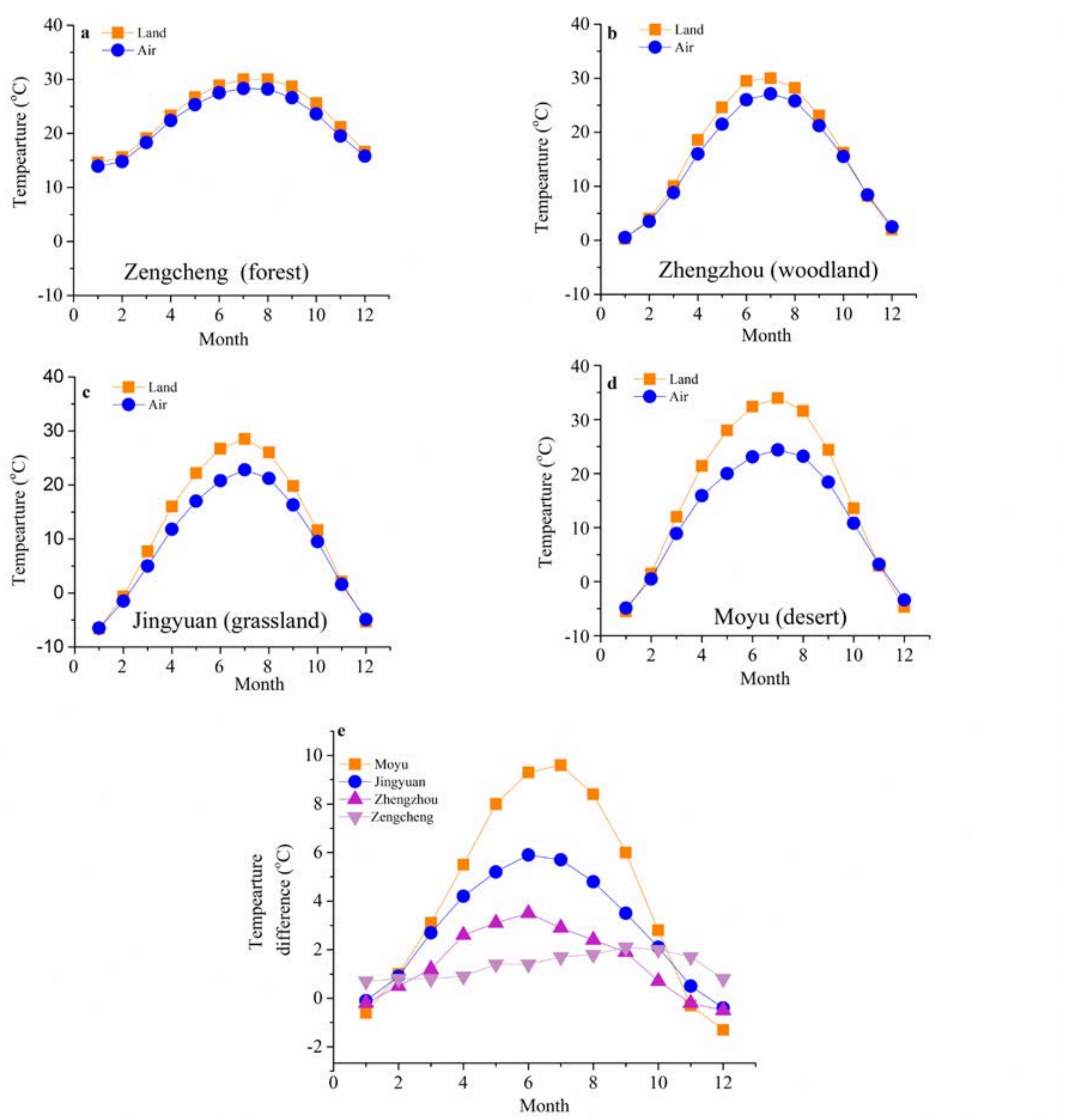

Supplementary Figure 3 Monthly land surface and air temperatures, and the contrast between the two temperatures from four meteorological stations. These 4 stations represent 4 vegetation zones in China, from forest (Zengcheng), woodland (Zhengzhou), grassland (Jingyuan), and desert (Moyu). The temperature contrast enhances with reduced vegetation cover.

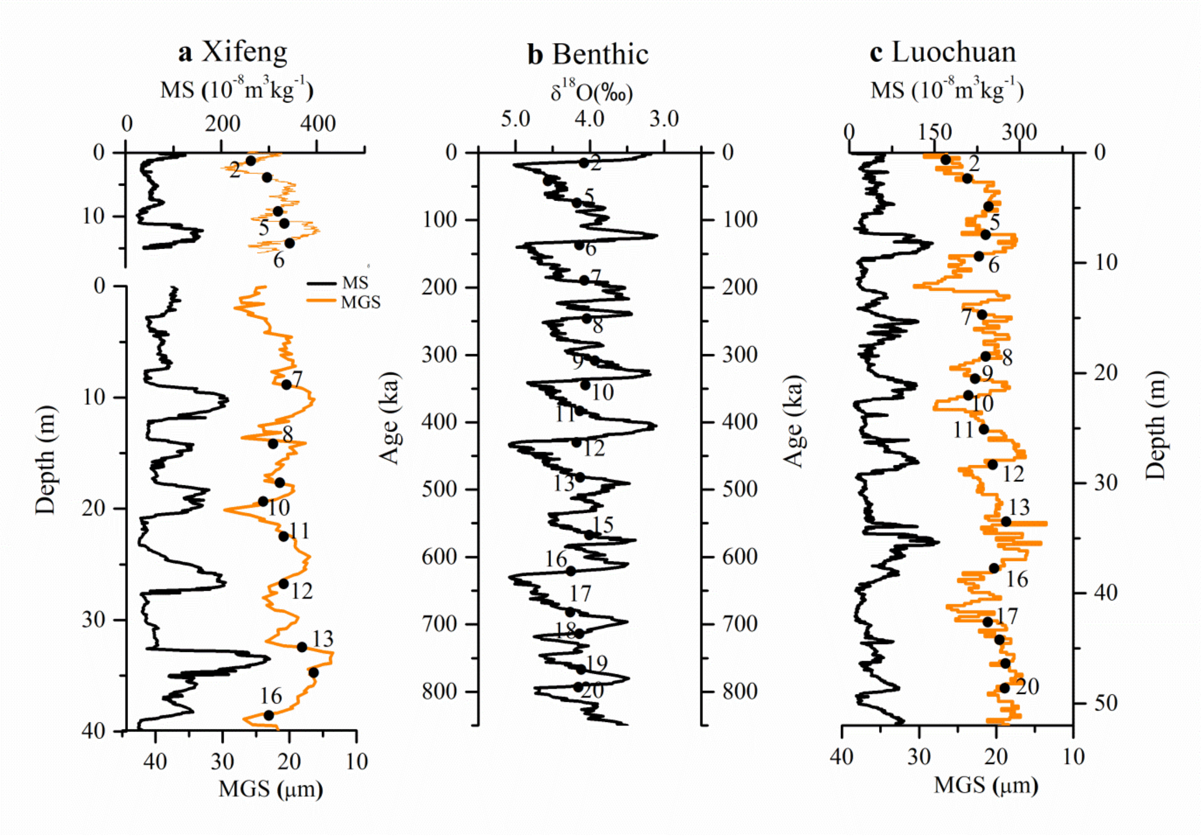

Supplementary Figure 4 Magnetic susceptibility and mean grain size variations in Xifeng and Luochuan sequences, used to derive a chronology, by correlating to the oxygen-isotope chronology. a Xifeng magnetic susceptibility (MS) (black) and mean grain size (MGS) (orange)**. b** Gloal benthic oxygen-isotope record[3]. **c** Luochuan MS (black) and MGS (orange)**.** Solid circles mark the mid-points of transitions between marine isotope stages and their proposed time equivalents in loess sequences.

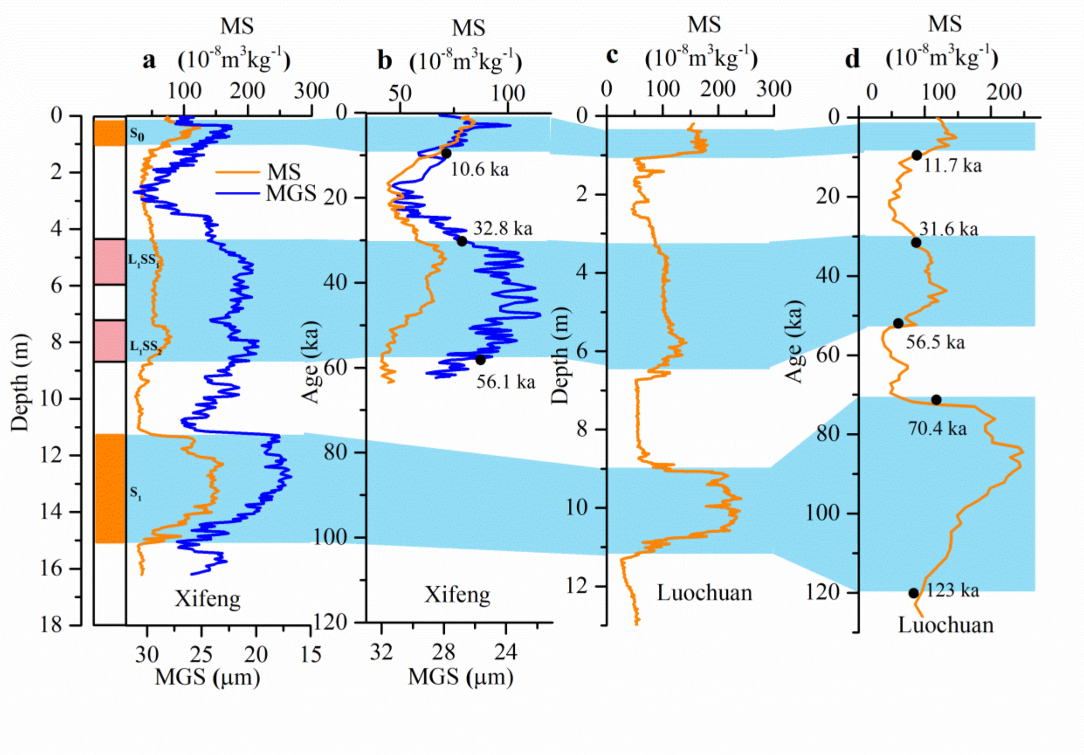

Supplementary Figure 5 Stratigraphy and magnetic susceptibility (MS) (orange) and mean grain size (MGS) (blue) variations at loess-paleosol sequences. a Xifeng (this study). **b** Xifeng[4]. **c** Luochuan (this study). **d** Luochuan[5]. Loess and paleosol units are shown to the right of the stratigraphic column. An alternative chronology at Xifeng[4] and Luochuan[5] sections over the past 130 kyr has been independently dated with optically stimulated luminescence technology, which is transferred to our study sections by correlating the loess–paleosol stratigraphy and MGS/MS variations.

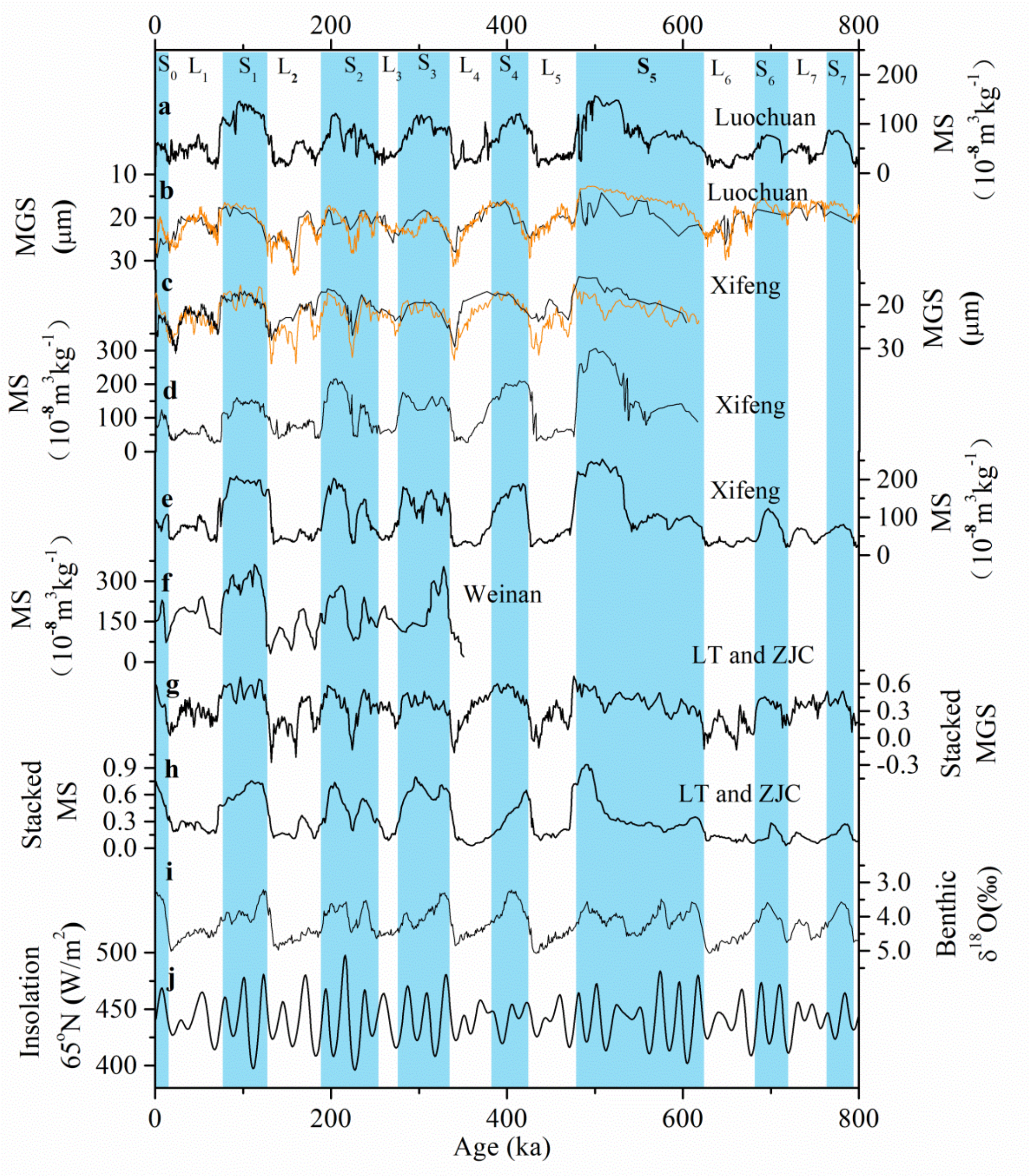

Supplementary Figure 6 Magnetic susceptibility and mean grain size variations at Xifeng and Luochuan sections compared with those from other loess-paleosol sequences on the CLP, as well as benthic $\delta^{18}O$ stack and insolation at 65°N over the past 800 kyr. a Magnetic susceptibility (MS) at Luochuan (this study). **b** Mean grain size (MGS) at Luochuan (black, this study; orange[6]). **c** MGS at Xifeng (black, this study; orange[7]). **d** MS at Xifeng (this study). **e** MS at Xifeng[8]. **f** MS at Weinan[9]. **g** Stacked MGS from Lingtai (LT) and Zhaojiachuan (ZJC)[7]. **h** Stacked MS from LT and ZJC[7]. **i** Benthic $\delta^{18}O$ stack[3]. **j** Summer (July 21) insolation at 65°N[10]. Highlighted are the major paleosol units and their correlation with benthic $\delta^{18}O$ and insolation.

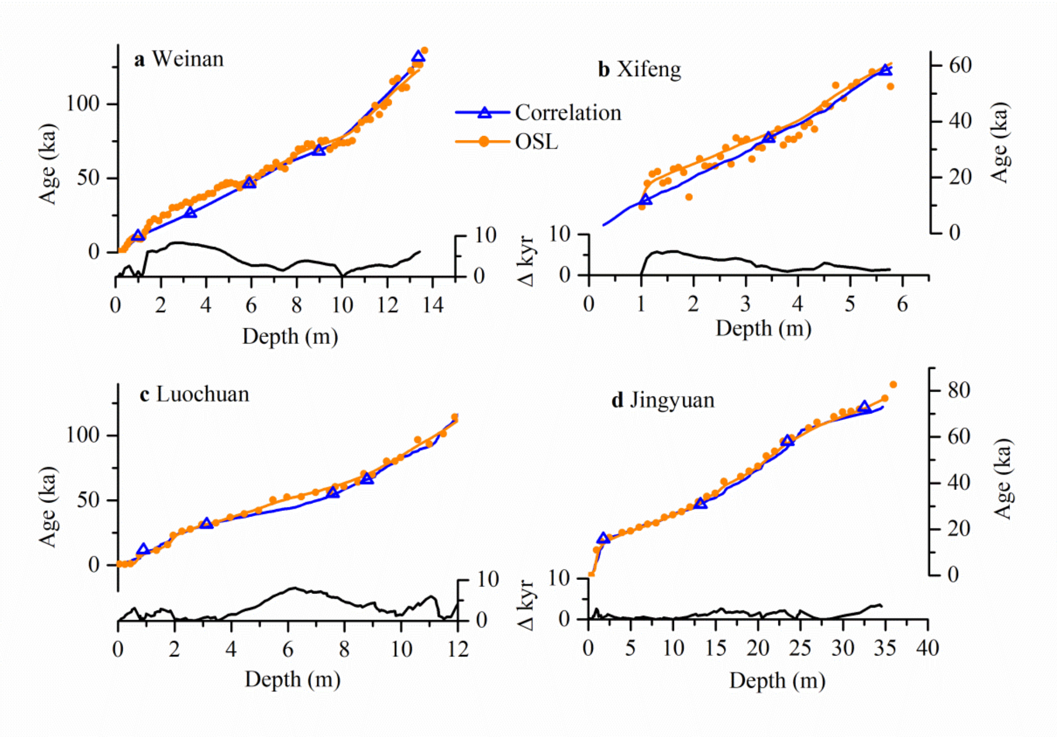

Supplementary Figure 7 Comparison of correlation-based with quartz optically stimulated luminescence derived chronologies over the past 60/130 kyr at four loess-paleosol sequences on the Chinese Loess Plateau. **a** Weinan[11]. **b** Xifeng[4]. **c** Luochuan[5]. **d** Jingyuan[12]. Orange dots denote optically stimulated luminescence (OSL) dates, while blue hollow triangles represent control points for the magnetic susceptibility/mean grain size age model. We used the BACON software[13] to derive the OSL-based chronology. The difference between the two chronologies is generally within 5 kyr, and less than 10 kyr in extreme cases.

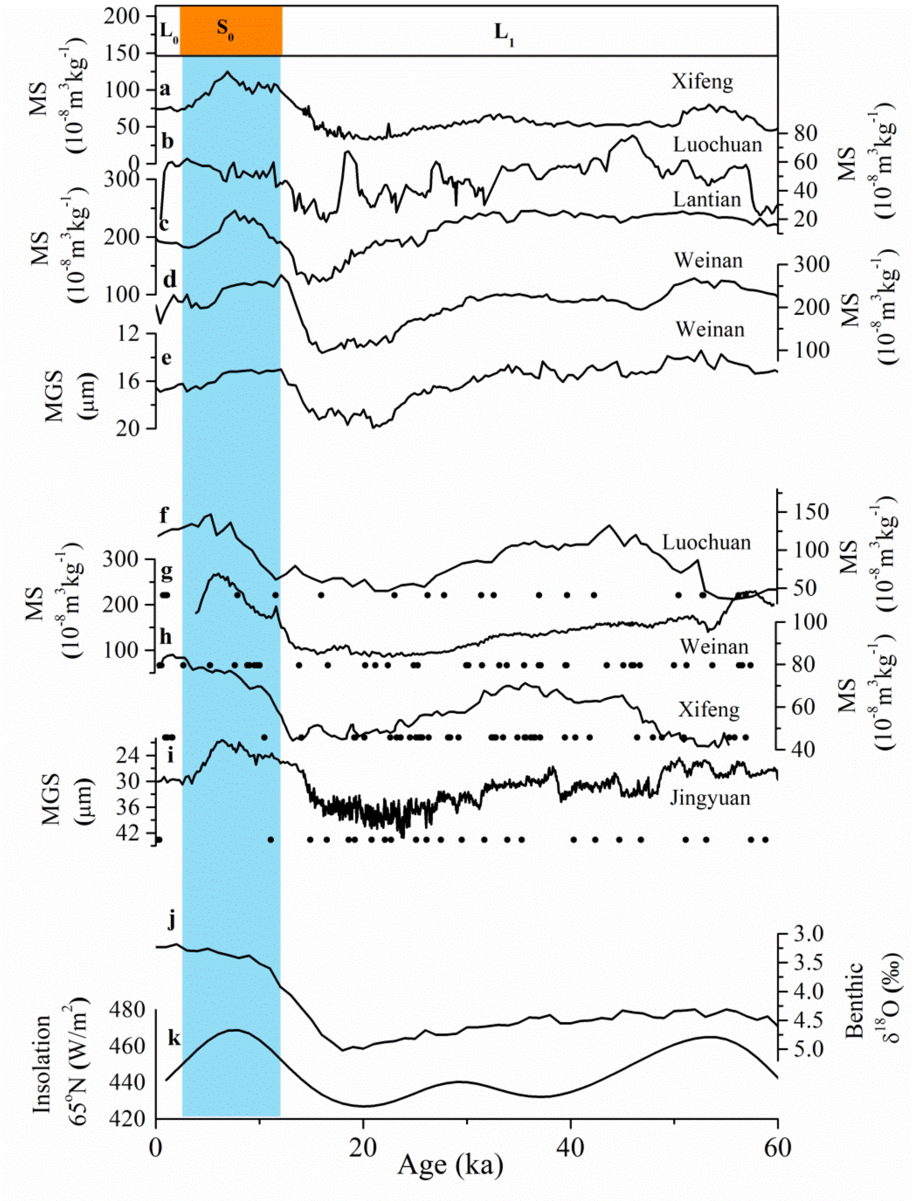

Supplementary Figure 8 Magnetic susceptibility / mean grain size time series, derived from correlation- or OSL-based chronologies, in loess-paleosol sequences on the Chinese Loess Plateau, compared with benthic $\delta^{18}O$ stack and insolation at 65°N over the past 60 kyr. **a** Xifeng magnetic susceptibility (MS). **b** Luochuan MS. **c** Lantian MS[14]. **d** Weinan MS[12]. **e** Weinan mean grain size (MGS) [12]. **f** Luochuan MS[5]. **g** Weinan MS[11]. **h** Xifeng MS[4]. **i** Jingyuan MGS[12]. **j** Benthic $\delta^{18}O$ stack[3]. **k** Summer (July 21) insolation at 65°N[10]. The blue shaded area indicates the major paleosol unit S_0. Black dots denote OSL dates. MS/MGS transitions, based on correlation to benthic $\delta^{18}O$ in **a-e** or OSL dating in **f-i**, typically fall in the interval 10-20 ka.

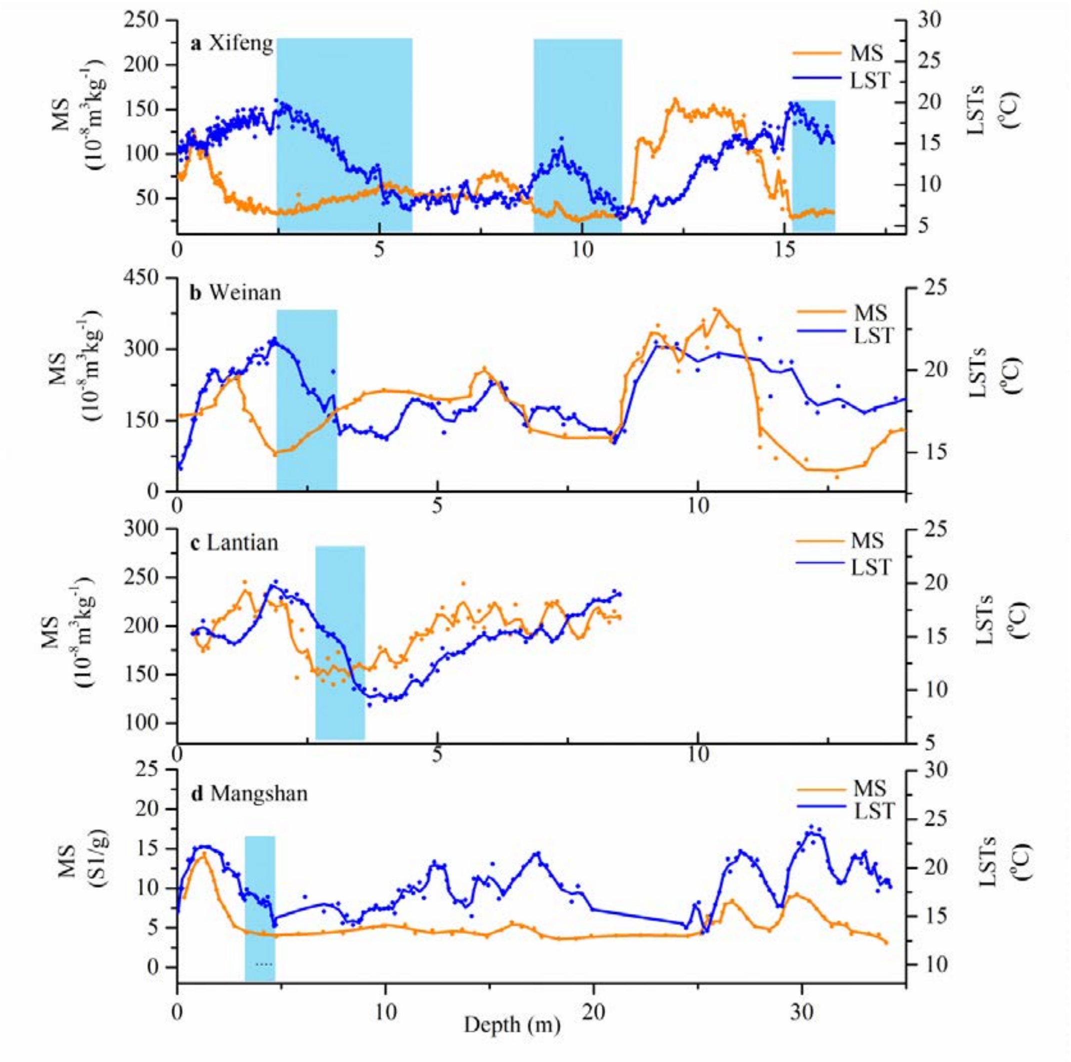

Supplementary Figure 9 Offset in depth between the land surface temperature (LST) rise and magnetic susceptibility (MS) changes in loess-paleosol sequences on the Chinese Loess Plateau. Offset in depth is highlighted with blue bars. The offset becomes larger from southeast (Mangshan) to northwest (Xifeng). See Fig. 1 for locations.

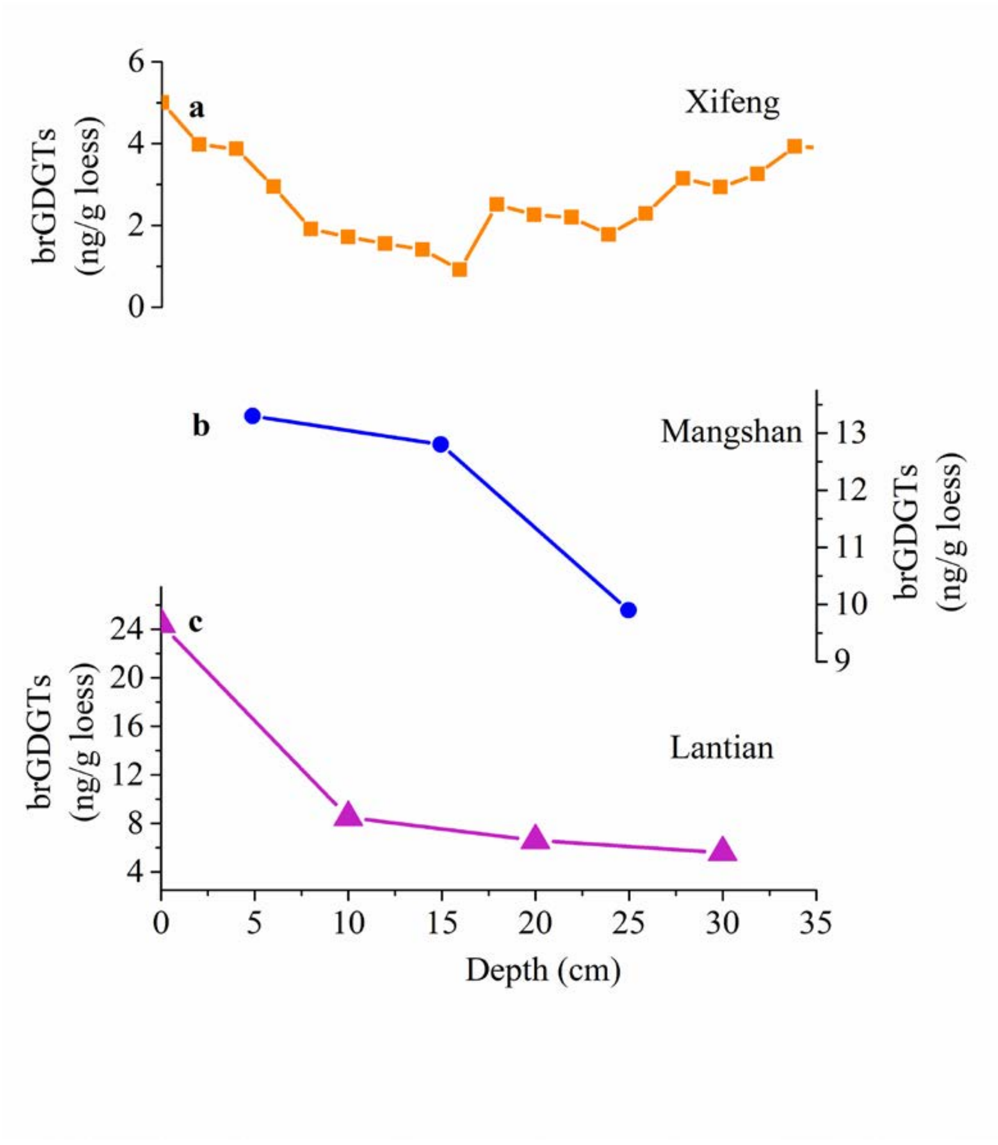

Supplementary Figure 10 Downcore variations of branched glycerol dialkyl glycerol tetraethers (brGDGT) contents in top soil/loess sequences. a Xifeng (this study). **b** Mangshan[15]. **c** Lantian[16]. High concentrations at top 5-10 cm suggest modern *in situ* production of brGDGTs.

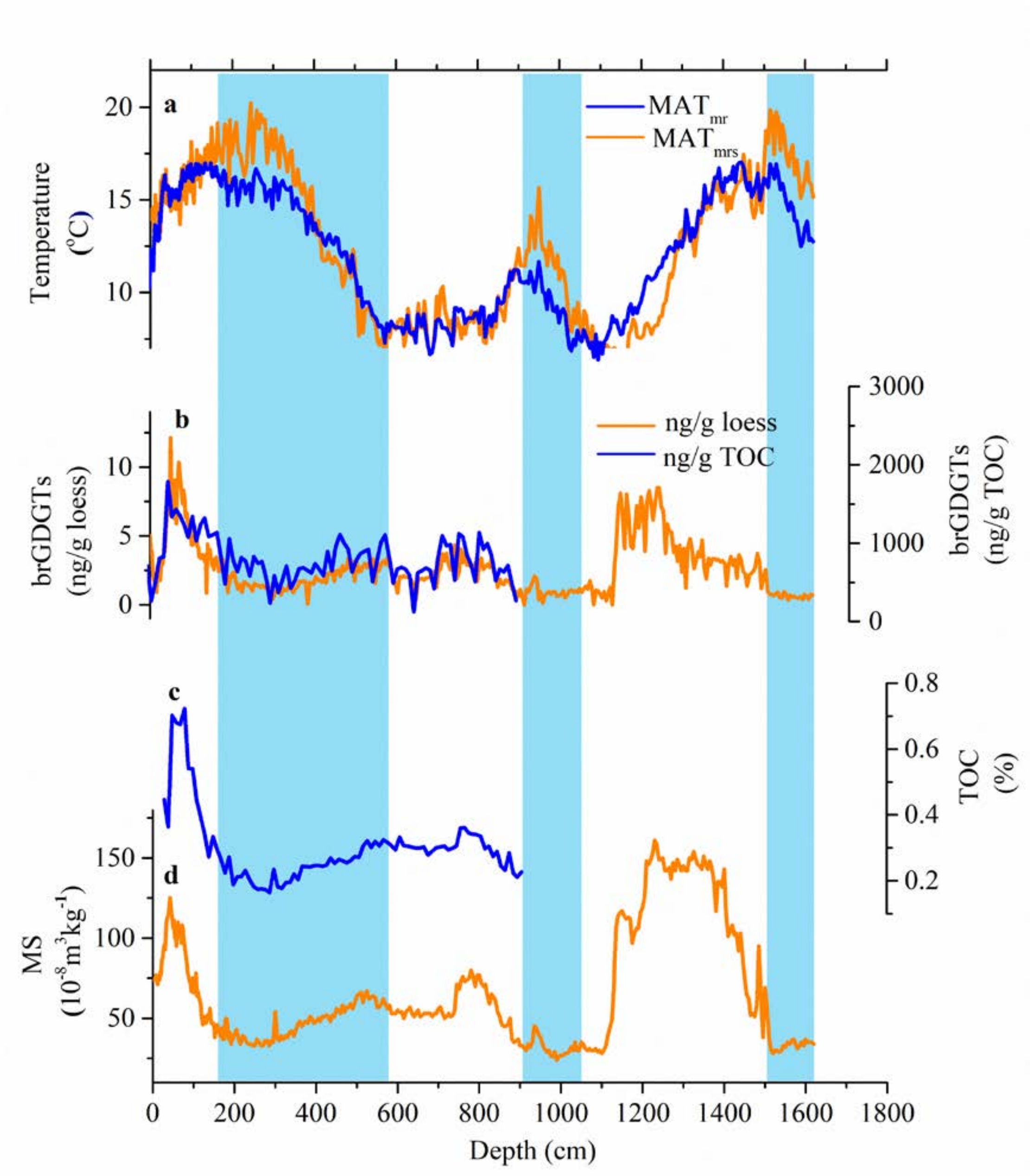

Supplementary Figure 11 Comparisons of branched glycerol dialkyl glycerol tetraethers based temperatures with other onsite geochemical indicators at the Xifeng section. a Land surface temperature. **b** Branched glycerol dialkyl glycerol tetraethers (brGDGT) concentrations. **c** Total organic carbon (TOC) contents. **d** Magnetic susceptibility (MS). The blue shaded areas indicate the period of LST rise, while brGDGT and TOC contents remained low.

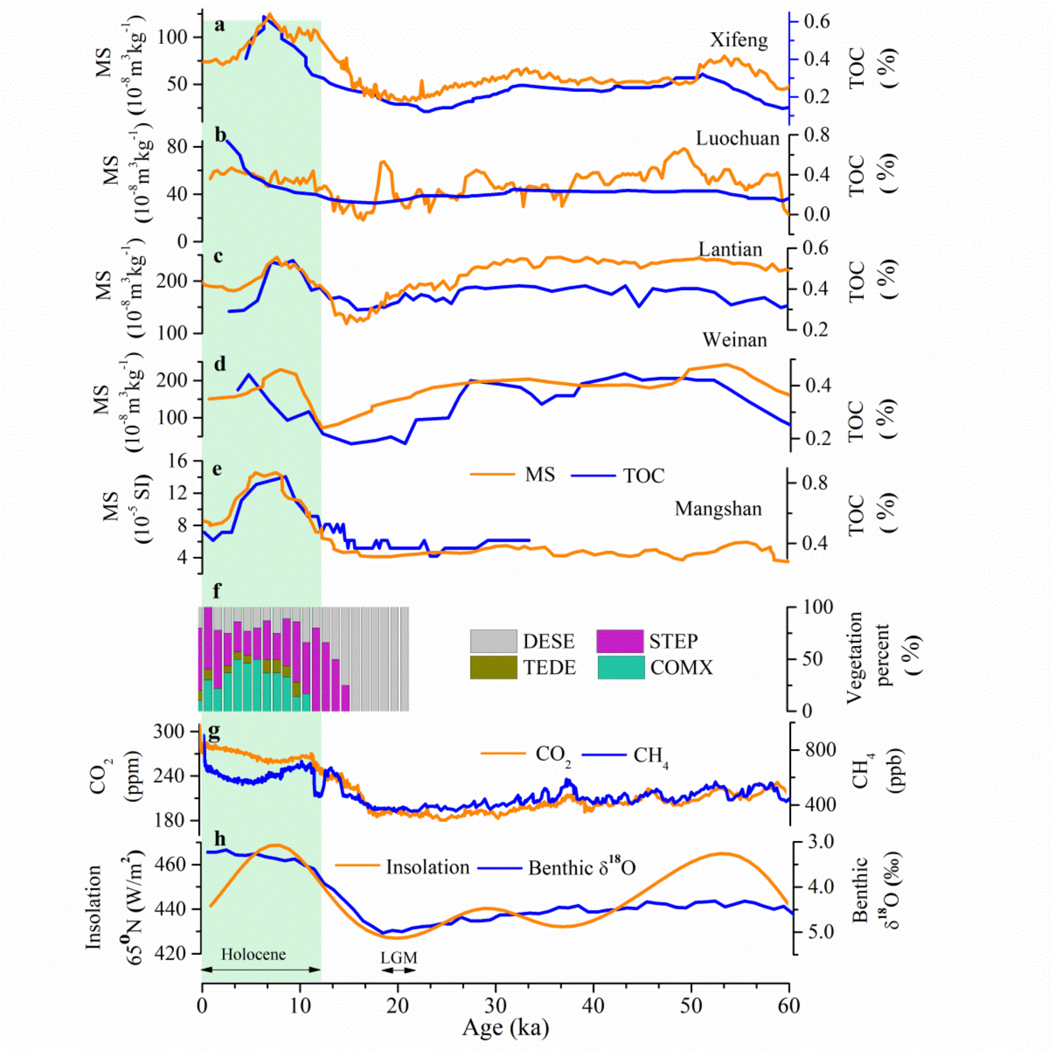

Supplementary Figure 12 Magnetic susceptibility (MS) and total organic carbon (TOC) in loess-paleosol sections, compared with reconstructed vegetation in northern China and global/marine records over the past 60 kyr. a Xifeng. **b** Luochuan. **c** Lantian. **d** Weinan[17]. **e** Mangshan[18]. **f** Vegetation changes in the East Sandy land of northern China[19] (COMX, cool mixed forest; TEDE, temperate deciduous forest; STEP, steppe; DESE, desert)**. g** CO_2 and CH_4 from Antarctic EDC ice core[20,21]. **h** Summer (July 21) insolation at 65°N[10] and benthic $\delta^{18}O$ stack[3]. TOC values at Mangshan were obtained by dividing the total organic matter value by a conventional conversion factor of 1.9, based on ref. 22. All the records show major changes during the deglacial period, 10-20 ka.

References

1. De Jonge, C. et al. Occurrence and abundance of 6-methyl branched glycerol dialkyl glycerol tetraethers in soils: Implications for palaeoclimate reconstruction. *Geochim. Cosmochim. Acta* **141**, 97-112 (2014).

2. Rodriguez, L. G. & Perez, M. R. Recent changes in Chinese forestry seen through the lens of Forest Transition theory. *Int. Forest Rev.* **15**, 456-470 (2013).

3. Lisiecki, L. E. & Raymo, M. E. A Pliocene-Pleistocene stack of 57 globally distributed benthic $\delta^{18}O$ records. *Paleoceanography* **20**, PA1003 (2005).

4. Stevens, T. *et al.* Mass accumulation rate and monsoon records from Xifeng, Chinese Loess Plateau, based on a luminescence age model. *J. Quat. Sci.* **31**, 391-405 (2016).

5. Lu, Y. C., Wang, X. L. & Wintle, A. G. A new OSL chronology for dust accumulation in the last 130,000 yr for the Chinese Loess Plateau. *Quat. Res.* **67**, 152-160 (2007).

6. Hao, Q. et al. Delayed build-up of Arctic ice sheets during 400,000-year minima in insolation variability. *Nature* **490**, 393-396 (2012).

7. Sun, Y., Clemens, S. C., An, Z. & Yu, Z. Astronomical timescale and palaeoclimatic implication of stacked 3.6-Myr monsoon records from the Chinese Loess Plateau. *Quat. Sci. Rev.* **25**, 33-48 (2006).

8. Guo, Z., Berger, A., Yin, Q. & Qin, L. Strong asymmetry of hemispheric climates during MIS-13 inferred from correlating China loess and Antarctica ice records. *Clim. Past* **5**, 21-31 (2009).

9. Tang, C. et al. Tropical and high latitude forcing of enhanced megadroughts in Northern China during the last four terminations. *Earth Planet. Sci. Lett.* **479**, 98-107 (2017).

10. Huybers, P. Early Pleistocene glacial cycles and the integrated summer insolation forcing. *Science* **313**, 508-511 (2006).

11. Kang, S. G., Wang, X. L. & Lu, Y. C. Quartz OSL chronology and dust accumulation rate changes since the Last Glacial at Weinan on the southeastern Chinese Loess Plateau. *Boreas* **42**, 815-829 (2013).

12. Sun, Y. B., Wang, X. L., Liu, Q. S. & Clemens, S. C. Impacts of post-depositional processes on rapid monsoon signals recorded by the last glacial loess deposits of northern China. *Earth Planet. Sci. Lett.* **289**, 171-179 (2010).

13. Blaauw, M. & Christen, J. A. Flexible Paleoclimate Age-Depth Models Using an Autoregressive Gamma Process. *Bayesian Anal.* **6**, 457-474 (2011).

14. Gao, L. et al. The importance of solar insolation on the temperature variations for the past 110 kyr on the Chinese Loess Plateau. *Palaeogeogr. Palaeoclimatol. Palaeoecol.* **317**, 128-133 (2012).

15. Peterse, F. et al. Molecular records of continental air temperature and monsoon precipitation variability in East Asia spanning the past 130,000 years. *Quat. Sci. Rev.* **83**, 76-82 (2014).

16. Lu, H., Liu, W., Wang, H. & Wang, Z. Variation in 6-methyl branched glycerol dialkyl glycerol tetraethers in Lantian loess-paleosol sequence and effect on paleotemperature reconstruction. *Org. Geochem.* **100**, 10-17 (2016).

17. Yang, H. et al. Correlations between microbial tetraether lipids and environmental variables in Chinese soils: optimizing the paleo-reconstructions in semi-arid and arid regions. *Geochim. Cosmochim. Acta* **126**, 49-69 (2014).

18. Peterse, F. et al. Decoupled warming and monsoon precipitation in East Asia over the last deglaciation. *Earth Planet. Sci. Lett.* **301**, 256-264 (2011).

19. Li, Q. et al. Reconstructed moisture evolution of the deserts in northern China since the Last Glacial Maximum and its implications for the East Asian Summer Monsoon. *Glob. Planet. Change* **121**, 101-112 (2014).

20. Bereiter, B. et al. Revision of the EPICA Dome C CO_2 record from 800 to 600 kyr before present. *Geophys. Res. Lett.* **42**, 542-549 (2015).

21. Loulergue, L. et al. Orbital and millennial-scale features of atmospheric CH_4 over the past 800,000 years. *Nature* **453**, 383-386 (2008).

22. Pribyl, D. W. A critical review of the conventional SOC to SOM conversion factor. *Geoderma* **156**, 75-83 (2010).
